# Influence of Breast Implant Surface Finishing on Physicochemical and Mechanical Properties before and after Extreme Degradation Studies

**DOI:** 10.1155/2021/8850577

**Published:** 2021-06-29

**Authors:** Izabelle de Mello Gindri, Lucas Kurth de Azambuja, Michele da Silva Barreto, Dionatha José do Prado, Gean Vitor Salmoria, Carlos Rodrigo de Mello Roesler

**Affiliations:** ^1^Laboratório de Engenharia Biomecânica, Hospital Universitário, Universidade Federal de Santa Catarina, Florianópolis, Santa Catarina, Brazil; ^2^Nimma, Núcleo de Inovação Em Moldagem e Manufatura Aditiva, Departamento de Engenharia Mecânica, Universidade Federal de Santa Catarina, Florianópolis, Santa Catarina, Brazil

## Abstract

The influence of the surface finishing of breast implants on physicochemical and mechanical properties, before and after extreme degradation experiments, was investigated in this study. Removal of superficial layers after degradation was verified for both smooth and rough membranes, in which local erosion was verified. FTIR results demonstrated the generation of low-molecular-weight structures in all samples due to exposure to acidic and basic environments. Furthermore, smooth samples presented higher degrees of crosslinking than rough samples. Considering the mechanical properties, no difference was verified between smooth and rough samples as received and after degradation studies. However, the pH of the degradation solution had an influence on mechanical properties of the material and a basic environment caused greater deterioration of the mechanical properties compared to acidic conditions.

## 1. Introduction

Breast implants have been widely employed for cosmetic and reconstructive surgeries since their invention by Cronin and Gerow in the 1960s [1]. During the following decades, there were changes in the properties of the materials use as well as in the prosthesis design. First-generation implants were manufactured as a thick shell filled with viscous silicone gels, generating very resistant devices [2, 3]. Despite the low rupture rates, after 10 years of use, almost 100% of these devices presented capsular contracture and calcification due to the implant features. To overcome this problem, the second generation of breast implants presented a new combination of materials consisting of a thin shell and less viscous filler, which could be silicone or saline solution. However, these devices presented rupture rates up to 60% [1] and leaking of filler fluid into the periprosthetic capsule, also defined as silicone “bleeding,” was frequently detected. The third generation consisted of a more durable, multilayered shell with a middle barrier layer that significantly reduced rupture and silicone bleeding. Also, the silicone filler contained larger particle size and increased crosslinking density to decrease material diffusion through the implant membrane. Since then, fourth-generation and fifth-generation implants have been introduced into the market, which are the implants currently in use. These devices have thicker shells combined with a more cohesive gel filler and are manufactured in smooth and textured shell models [1–3].

Recent studies on breast implant rupture rates indicate values of 0 to 17.7%. This may occur in the case of primary augmentation, revision augmentation, primary reconstruction, or revision reconstruction. Rupture mechanisms include shell swelling, fold flaw, damage from surgical instruments, or trauma to the implant [4–6].

The shell surface topography influences the implant performance in both early and late stages after implantation. Smooth surfaces are historically associated with capsular contracture, which is the tightening and hardening of the normal capsule that encases the breast implant [1]. This process results in pain, poor aesthetic appearance, reoperation, and ultimately patient dissatisfaction [1]. On the other hand, surface roughness directly increases the implant surface area and improves the host response after implantation. However, this surface feature has been associated with increased bacterial adhesion and anaplastic large cell lymphoma [7–9].

Several studies have been conducted to investigate the biological response towards smooth and rough surfaces, but the influence of surface treatment on the mechanical properties of implant shells has not been well explored. Persichetti et al. evaluated the influence of surface finishing on the chemical properties. Potentially reactive groups, known as silanols, were identified in all shells but were present in high intensity in textured implants [10].

The biochemical environment to which these devices are exposed is also an important parameter to understand their performance *in vivo*. Biological pH is known to vary during the wound-healing process. Percival et al. verified that wound-healing progression decreased under alkaline conditions [11]. Moreover, there is evidence that the acute and chronic wound environment progresses from an alkaline state to a neutral state and then an acidic state during healing [11]. However, few authors have investigated the effect of pH on the physicochemical and mechanical properties of breast implants. In a recent study, we investigated a series of implants from different manufacturers with regard to their chemical composition, thermal properties, and mechanical strength. The implants with rough surfaces were found to be more sensitive to acidic degradation [12].

This paper reports a study aimed at determining the influence of the surface features of breast implants on their physicochemical and mechanical properties after exposure to extreme acidic and basic degradation. An evaluation of the performance, using an approach proposed in our previous study, was conducted based on the morphological features, chemical composition, thermal properties, and mechanical strength of breast implants before and after degradation studies.

## 2. Experimental

### 2.1. Materials

Six pristine implants from the same manufacturer were selected for this study: three (*n* = 3) with smooth surfaces and three (*n* = 3) with rough surfaces. Each implant was separated into two parts, top and bottom of shell, and the filling gel was carefully removed. The membranes were cleaned with isopropyl alcohol PA (Dinâmica Química Contemporânea Ltda, Indaiatuba, Brazil). Samples with smooth and rough surfaces are also referred to herein as smooth and rough membranes, respectively. Both types of membranes were characterized before and after degradation studies.

### 2.2. Scanning Electron Microscopy and Energy Dispersive Spectroscopy

The specimens were collected from the bottom of each implant and cut into small squares of 2 mm × 2 mm. These were covered with gold and dried for 24 h in a dissector. They were then evaluated by scanning electron microscopy (SEM) using a JEOL JSM-6390LV scanning electron microscope (Akishima, Tokyo, Japan). The same samples used for the SEM were also used for the energy dispersive spectroscopy (EDS) conducted on an instrument coupled to the SEM microscope.

### 2.3. Fourier Transform Infrared Spectroscopy

Attenuated total reflectance-Fourier transform infrared (ATR-FTIR) spectra (obtained with 400–4000 cm^−1^, scans: *n* = 32, resolution = 4 cm^−1^) were recorded using a Perkin Elmer spectrometer (Waltham, Massachusetts, United States) equipped with an ATR unit (Pike GladiATR™ Vision). According to the technical standards ISO 14949 : 2018 (subsection 6.2) and ASTM 1252–9, the infrared spectra of silicone samples should show six characteristic absorption peaks: 2962 ± 5 cm^−1^ (-Si(CH_3_)_2_); 2906 ± 5 cm^−1^ (-Si(CH_3_)); 1260 ± 5 cm^−1^ (-Si(CH_3_)_2_); 1094 ± 5 cm^−1^ (-Si(CH_3_)_2_-O-Si(CH_3_)_2_); 1022 ± 5 cm^−1^ (-Si-O-Si-) 10; and 765 ± 5 cm^−1^ (CH_3_). For FTIR analysis, 10 cm × 10 mm squares of each implant were obtained from the bottom of shell.

### 2.4. Differential Scanning Calorimetry

Differential scanning calorimetry (DSC) curves were recorded using a Perking Elmer 6000 (Waltham, Massachusetts, United States). Two specimens extracted from each implant shell were cut and weighed (approximately 7 mg ± 1 mg), and the weight measurement was done in a Shimadzu digital balance with 0.001 g precision. The specimens were placed in aluminum pans, which were then sealed. The analysis was conducted following the technical standards ISO 11357–1 (2016) and ASTM D3418 (2015) in an instrument supplied with ultrapure nitrogen gas with 19.8 ml/min flow. The method consisted of five steps: (i) 30°C for 3 min; (ii) decrease from 30°C to –90°C at 10°C/min; (iii) −90°C for 30 min; (iv) increase from –90°C to 30°C at 10°C/min; and (iv) 30°C for 3 min.

Crystallinity (*a*_*c*_) values were obtained by the following equation:

(1) $$\begin{array}{c} a_{c}\left(\%\right)=\frac{\Delta h}{\Delta h_{c}}\;\cdot 100. \end{array}$$

The value of enthalpy of the material analyzed (Δ*h*) was obtained by the integral of the fusion peak done by the function in the software Pike. The value used for the enthalpy of fusion of the 100% crystalline material (Δ*hc*) of polydimethylsiloxane used was 38.2 J/g [13].

### 2.5. Swell Test

Crosslinking values were obtained by swell test method based on the work produced by Di Kassia [14]. The 10 mm × 10 mm specimens were weighed, and the value was collected (Mo). Then, the specimens were immersed in xylene (analytical grade), for 24 hours at 40 ± 1°C. After this period, the samples were extracted from the xylene and dried in a vacuum chamber for extra 24 hours. The samples were weighed and put for extra 24 h dry. After 24 h, the specimens were weighed again, and if there was no difference between measures, the weight value (Ms) was collected. The weight measurement was done in a Shimadzu digital balance with 0.001 g precision. The crosslinking percentage was measured by the following equation:

(2) $$\begin{array}{c} \text{crosslinking}\left(\%\right)=\left(\frac{M_{s}}{M_{o}}\right)\times 100. \end{array}$$

### 2.6. Mechanical Test

The mechanical tests were conducted according to the technical standards ISO 14607:2018(E) [15] and ASTM D412 [16]. Four specimens (*n* = 4) of each implant were extracted in a tie shape according to ISO 37:20172 [17] and the thickness was measured with a digital micrometer (Supplementary Information). Experiments were conducted on a universal testing machine EMIC DL3000 with a 50 kfg load cell (model EMIC-SV50). Each specimen was attached at the extremity, between two claws, with a clip gage displacement transducer (model EMIC EE04) installed in the central portion of the specimen. The tests were conducted in displacement control mode at 500 mm/min rate, with a preload of 0.2 N, and the force (N) versus displacement (mm) curve was obtained. The stress-strain curves were calculated considering the specimen's transversal cross-sectional area (mm^2^) and the initial gage length.

### 2.7. Degradation

Smooth and rough membranes were exposed to a degradation environment for 90 days at 37.5°C. The acidic solution consisted of hydrochloric acid (pH of 1.25) and the basic solution was composed of sodium hydroxide (pH 13). Solution pH was measured using a PEG Tecnopon with an Ag/AgCl cell. For this analysis, 10 cm × 10 mm squares obtained of each implant were extracted from the bottom of shell of the implant.

### 2.8. Statistical Analysis

To evaluate the effect of two variables (degradation process and roughness) over the answer variables (deformation on the rupture, strain on 450% of deformation, and strain on the rupture), an analysis of variance with two factors was applied, followed by Tukey's test for paired comparisons. A log transformation of the data was done when the conditions of variance homogeneity were not satisfied. The level of accuracy of 0.05 was used.

## 3. Results and Discussion

The lifetime of breast implants is still a matter of intense interest and debate among the plastic surgery community as well as among the patients. The effect of surface finishing has been evaluated in terms of biological activity, in which smooth surfaces were associated with capsular contracture and rough surfaces with bacterial adhesion and lymphomas. In addition, the evaluation of the chemical properties demonstrated higher hydrophilic character in rough than in smooth breast implant samples [10]. However, the effect of surface finishing on the mechanical properties and sample sensitivity towards degradation has not been investigated. In this study, a systematic evaluation was conducted employing a methodology recently proposed by our group.

**Figure 1:**
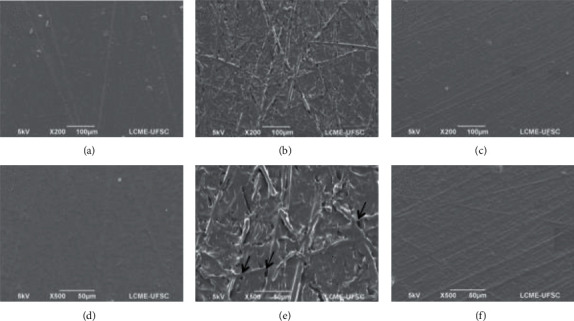
SEM images of smooth membranes at 200 X: (a) as-received, (b) after acidic degradation, and (c) after basic degradation; and at 500 X: (d) as-received, (e) after acidic degradation, and (f) after basic degradation.

**Figure 2:**
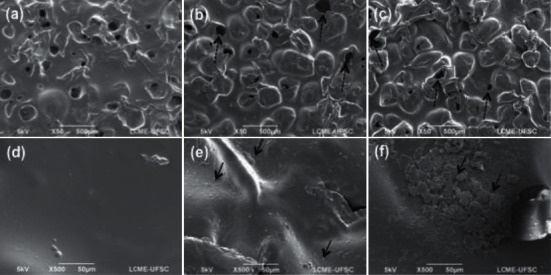
SEM images of rough membranes at 200 X: (a) as-received, (b) after acidic degradation, and (c) after basic degradation; and at 500 X: (d) as-received, (e) after acidic degradation, and (f) after basic degradation.

Microscopic characteristics of smooth and rough implants examined in this study are demonstrated in Figures 1 and 2, respectively. Before degradation (Figure 1(a)), the smooth samples had discreet lines on the surface which result from the manufacturing process (Figure 1(a)). After acidic degradation (Figures 1(b) and 1(c)), the manufacturing marks became more evident, and the formation of pits could be observed as indicated by black arrows. The basic solution also caused changes in the surface features, and the machine marks became more evident compared with the as-received sample, as shown in Figures 1(c) and 1(f).

Rough implants presented irregular surfaces, and changes were verified after basic and acid degradation. The pristine rough surface has features such as peaks, valleys, and pit-like structures, which are formed during the manufacturing process (Figure 2(a)). Despite these irregularities, the membrane as well as the pit edges had a smooth finish, as evidenced by the magnification of a valley surface in Figure 2(d). On the other hand, after acid (Figures 2(b) and 2(e)) and basic (Figures 2(c) and 2(f)) degradation, the membrane surfaces had more irregularities. Erosion features (black arrows) were observed on all samples exposed to degradation conditions. Furthermore, the pit edges suffered a sharpening process as indicated by the dashed arrows.

In summary, the SEM images demonstrated surface deterioration after basic and acidic degradation periods. The erosion of superficial layers resulted in a more irregular surface for both smooth and rough samples. Changes in pit morphology were also verified for the rough samples, where the pit edges became sharper after immersion in the degradation solutions. These results are in accordance with a study performed by Amin et al. where similar features were observed when PDMS samples were exposed to environmental conditions [18].

**Figure 3:**
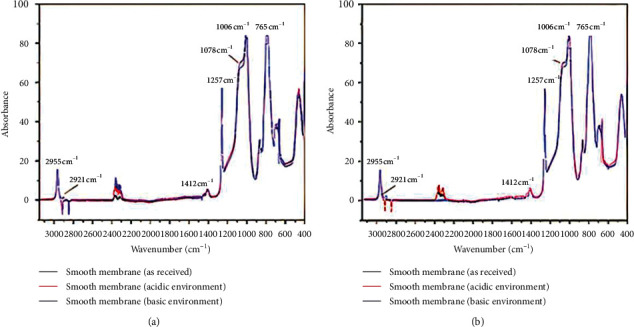
FTIR spectra for (a) smooth and (b) rough membranes before and after degradation studies.

**Figure 4:**
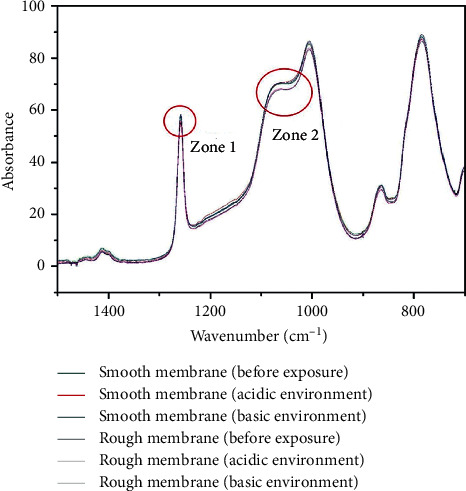
Magnified FTIR spectra for sample A in the range of 1500–700 cm^−1^. Zone 1 shows a zoom of the normalized peak around 1257 cm^−1^ and Zone 2 shows a zoom of the region around 1084 cm^−1^.

The morphological changes are consistent with alterations in the chemical structure, as demonstrated by the sample compositions determined using FTIR before and after the degradation studies. The spectra for the smooth and rough samples, before and after degradation, are shown in Figures 3(a) and 3(b), respectively. Characteristic PDMS peaks were identified for all samples at 2955 cm^−1^ (CH in CH_3_), 2921 cm^−1^ (CH3), 1456 cm^−1^ (CH_3_ with asymmetrical deformation), 1412 cm^−1^ (CH_2_), 1257 cm^−1^ (CH_3_ with asymmetrical deformation), 1078 cm^−1^ (Si-O-Si), 1006 cm^−1^ (-Si(CH_3_)_2_-OSi(CH_3_)_2_), and 765 cm^−1^ (Si-(CH_3_)_2_). After basic and acidic degradation, both the rough and smooth samples presented increased intensity at 1257 cm^−1^ and 1078 cm^−1^, which indicates the formation of small-molecular-weight siloxanes during the degradation process (Figure 4) [19]. An increase in low-molecular-weight polymers has been previously detected using FTIR by Yildirimer et al. [20] as well as in a recent study by our group [12].

Thermal behavior was also employed as a monitoring tool to evaluate changes in the materials before and after degradation. This important tool can be used to investigate the chemical structure, since the molecular weight influences the melting point and polymer crystallinity [3].

**Figure 5:**
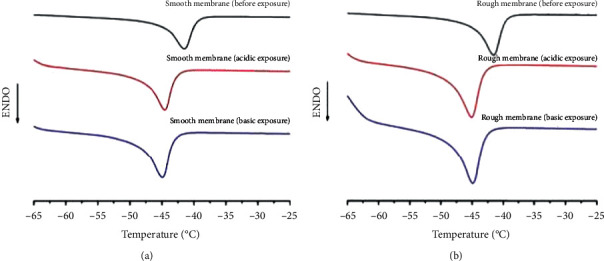
DSC curves for (a) smooth and (b) rough membranes.

DSC curves and thermal properties are shown in Figure 5 and Table 1, respectively. A reduction in the melting point was observed after acidic and basic degradation for both smooth and rough membranes, which indicates the formation of low-molecular-weight structures [3]. Previous studies have demonstrated that melting temperature is proportional to molecular weight and the crystallization of longer polymeric chains results in higher melting points [21]. Therefore, the presence of shorter PDMS molecules in sample structures after degradation explains the reduction in the melting point. Smooth samples also presented increased melting enthalpy after degradation, while this parameter remained similar before and after degradation for rough samples.

Smooth membranes presented lower crystallinity values compared to rough membranes, for the as-received samples as well as after degradation studies. Lower crystallinity is associated with a higher degree of crosslinking, as reported by Roland and Aroson [22]. An increase in crystallinity was also observed for samples after degradation. A greater difference between values for the as-received samples and after degradation was observed for the smooth membranes. According to our previous findings, degradation is more likely to occur in crystalline (non-cross-linked) regions, corroborating the results found in this study [12].

**Figure 6:**
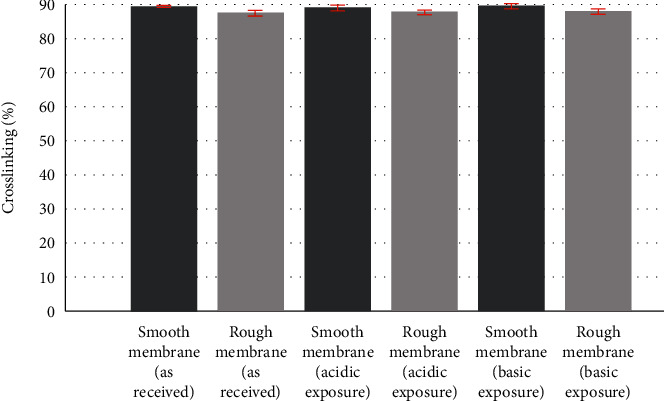
Percentage of crosslinking for smooth and rough breast implant membranes.

The crosslinking percentage is shown in Figure 6 and Table 2 for all samples. Results demonstrated that smooth samples have higher crosslinking density compared to rough samples before and after degradation studies. However, no change was observed within the groups of smooth membranes or rough membranes after the degradation studies.

The results for the mechanical properties of the samples as received and at the end of degradation experiments are shown in Table 3 and Figures S2–S7 (Supplementary Information). All as-received samples fulfilled the criteria of ISO 14607 and deformed by 450% without failures. The data show similar mechanical strength for the smooth and rough membranes and for the samples as received and after degradation studies. Considering the chemical degradation, both smooth and rough membranes were susceptible to mechanical deterioration under basic conditions. This behavior corroborates the findings of previous studies in which basic conditions caused higher degradation in PDMS samples in comparison to acidic conditions [23]. According to Hamilton, in basic catalysis, the silicon atom behaves as an electrophilic site for the nucleophilic attachment of hydroxyl groups. On the other hand, acid-catalyzed hydrolysis is initiated by oxygen protonation, which makes the carbon more electrophilic and thus more susceptible to chemical degradation. Due to the random protonation of the siloxane oxygen under acidic conditions, there is the formation of less reaction sites under acidic conditions in comparison to the basic environment, which explains the results obtained in this study [24].

An ANOVA test was done to evaluate the effect of two variables, degradation process and roughness, over the mechanical strength (MPa) at 450% strain, mechanical strength (MPa) at rupture, and deformation at rupture (Table 4). The results show that roughness and roughness associated with degradation process do not interfere in the mechanical properties. However, degradation process interferes in the mechanical properties of the material.

A Tukey test evaluated the influence of the degradation process over mechanical strength (MPa) at 450% strain and mechanical strength (MPa) at rupture. The results are shown in Table 5.

Tukey's test results presented in Table 5 confirmed that there is a difference between groups analyzed. Strain on 450% of deformation and strain on the rupture showed difference only when comparing groups as received and exposure to basic solution. Deformation showed a difference between groups as received with acidic explosion and with basic explosion.

The durability and useful life of a breast implant continue to be a subject of intense interest and debate among researchers, patients, and the plastic surgery community. Recent studies have demonstrated the potential for the application of novel technologies to breast implant surfaces, such as nanotexturization and graphene materials [25, 26]. Nanosurfaces can improve compatibility between implant and tissues, reducing inflammation and inflammation-related complications, such as capsular contracture, double capsules, and late seromas. On the other hand, graphene has good potential for application in breast implant texturization, since this technology has demonstrated antimicrobial affects and can extend the lifetime of biomaterials.

## 4. Conclusions

The influence of surface finishing on physicochemical and mechanical properties of breast implants, before and after extreme degradation experiments, was investigated. The SEM results showed differences in the sample surfaces before and after degradation, for both smooth and rough membranes. The removal of superficial layers associated with local erosion was verified. FTIR results demonstrated the generation of low-molecular-weight structures in all samples during exposure to acidic and basic environments. Furthermore, smooth samples presented a higher crosslinking degree compared to rough samples. However, in this study, the surface texture did not influence the mechanical properties of samples as received or after exposure to the degradation conditions. On the other hand, pH had a notable impact on the degradation profile, where a basic environment led to greater deterioration of the mechanical properties compared to acidic conditions. The use of extreme degradation conditions does not allow comparing the degradation profile with simulated body conditions; however, it allows the direct comparison of chemical resistance of different surface structures.

## Figures and Tables

**Table 1:** Thermal properties for samples as received and after acidic and basic degradation.

	Smooth membranes			Rough membranes		
	As received	Acidic exposure	Basic exposure	As received	Acidic exposure	Basic exposure
Melting temperature (C)	−41.5	−44.5	−45.0	−41.5	−45.2	−44.8
Enthalpy (J/g)	10.5	12.9	12.9	14.2	14.5	14.3
Crystallinity (*a*_*c*_) (%)	27.5	33.7	33.7	37.2	38.0	37.4

**Table 2:** Crosslinking for samples as received and after acidic and basic degradation.

	Smooth membranes			Rough membranes		
	As received	Acidic exposure	Basic exposure	As received	Acidic exposure	Basic exposure
Crosslinking (%)	89.5 ± 0.3	89.0 ± 0.7	89.7 ± 0.8	87.7 ± 0.5	87.8 ± 0.6	88.2 ± 0.8

**Table 3:** Mechanical properties for samples as received and after acidic and basic degradation.

Roughness	Smooth membranes			Rough membranes		
Degradation	As received	Acidic	Basic	As received	Acidic	Basic
Mechanical strength (MPa) at 450% strain^a^	6.7 ± 0.5	7.6 ± 1.3	4.9 ± 1.6	5.3 ± 0.6	7.3 ± 1.2	6.0 ± 1.5
Mechanical strength (MPa) at rupture	9.2 ± 0.9	8.1 ± 0.9	5.2 ± 1.9	7.9 ± 1.5	8.0 ± 0.5	6.7 ± 2.7
Deformation (%) at rupture	556 ± 61	465 ± 48	416 ± 66	539 ± 119	473 ± 51	396 ± 147

^a^Average of samples that reached 450% deformation.

**Table 4:** ANOVA test comparing degradation process and roughness over mechanical strength (MPa) at 450% strain, mechanical strength (MPa) at rupture, and deformation at rupture.

Property	Mechanical strength at rupture (MPa)	Mechanical strength at 450% strain (MPa)	Deformation at rupture (%)
Roughness	0.652	0.899	0.724
Degradation process	0.002	0.001	0.004
Degradation process and roughness	0.1373	0.102	0.758

**Table 5:** Tukey's test results from comparison of degradation process over mechanical properties.

Property	Comparison of degradation group process	*p* value
Mechanical strength (MPa) at 450% strain	Basic-acid	0.012
	As received-acid	0.076
	As received-basic	0.668

Mechanical strength (MPa) at rupture	Basic-acid	0.011
	As received-acid	0.882
	As received-basic	0.004

Deformation (%) at rupture	Basic-acid	0.195
	As received-Acid	0.048
	As received-basic	<0.001

## Data Availability

The data used to support the findings of this study are available upon request.

## Supplementary Materials

Supplementary Materials Sample figures and codification as well as mechanical graphs.
