# Supplementary material for: Influence of Breast Implant Surface Finishing on Physicochemical and Mechanical Properties before and after Extreme Degradation Studies
Source: Int J Biomater. 2021 Jun 29;2021:8850577. doi: 10.1155/2021/8850577 (PMC8261180; doi:10.1155/2021/8850577)
Supplement: Supplementary Materials — Sample figures and codification as well as mechanical graphs. [file 8850577.f1.docx]

**SUPPORTING INFORMATION**

**INFLUENCE OF BREAST IMPLANT SURFACE FINISHING ON PHYSICOCHEMICAL AND MECHANICAL PROPERTIES BEFORE AND AFTER DEGRADATION STUDIES**

Izabelle de Mello Gindri*^a^*, Lucas Kurth Azambuja*^a^*, Michele Barreto*^a^*, Dionatha José do Prado^a^, Gean Vitor Salmoria*^a,b^* Carlos Rodrigo de Mello Roesler*^a^*

^a^*Laboratório de Engenharia Biomecânica, Hospital Universitário, Universidade Federal de Santa Catarina, Florianópolis, Santa Catarina, Brasil,* [*r.roesler@ufsc.br*](mailto:r.roesler@ufsc.br)*,* <http://www.lebm.ufsc.br>

*^b^Nimma, Núcleo de Inovação em Moldagem e Manufatura Aditiva, Departamento de Engenharia Mecânica, Universidade Federal de Santa Catarina, Florianópolis, Santa Catarina, Brasil,* [*gean.salmoria@ufsc.br*](mailto:gean.salmoria@ufsc.br)


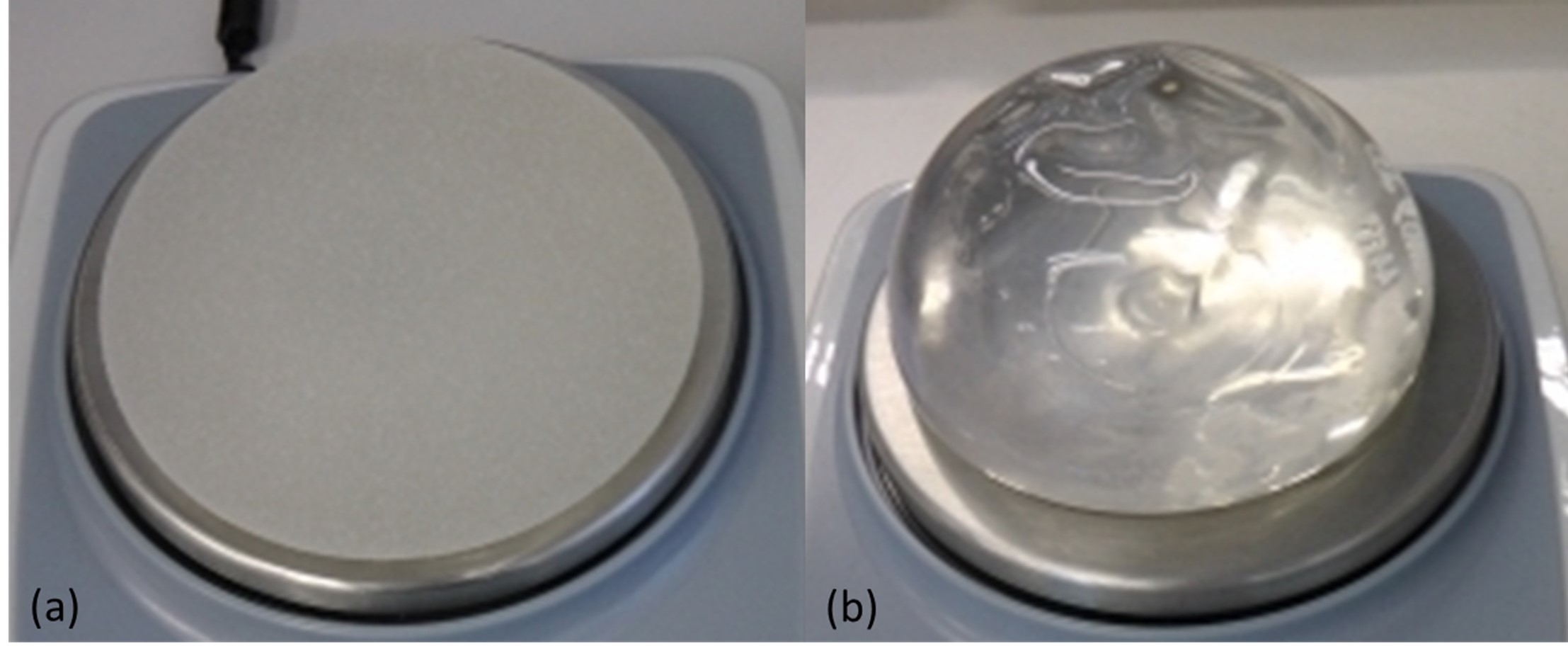


Figure S1. Representative images of the (a) rough and (b) smooth implants under investigation.

Table. 1: Mechanical Proof body summary with media thickness

| Sample |  | Thickness |
| --- | --- | --- |
| Smooth Membrane | 1S | 0,419 |
| Smooth Membrane | 2S | 0,425 |
| Smooth Membrane | 3S | 0,4 |
| Smooth Membrane | 4S | 0,342 |
| Rough Membrane | 1R | 0,539 |
| Rough Membrane | 2R | 0,55 |
| Rough Membrane | 3R | 0,515 |
| Rough Membrane | 4R | 0,532 |
| Smooth Membrane basic | 1SB | 0,465 |
| Smooth Membrane basic | 2SB | 0,516 |
| Smooth Membrane basic | 3SB | 0,598 |
| Smooth Membrane basic | 4SB | 0,556 |
| Rough Membrane basic | 1RB | 0,463 |
| Rough Membrane basic | 2RB | 0,387 |
| Rough Membrane basic | 3RB | 0,508 |
| Rough Membrane basic | 4RB | 0,555 |
| Smooth Membrane acidic | 1SA | 0,355 |
| Smooth Membrane acidic | 2SA | 0,453 |
| Smooth Membrane acidic | 3SA | 0,564 |
| Smooth Membrane acidic | 4SA | 0,55 |
| Rough Membrane acidic | 1RA | 0,471 |
| Rough Membrane acidic | 2RA | 0,418 |
| Rough Membrane acidic | 3RA | 0,571 |
| Rough Membrane acidic | 4RA | 0,603 |

Fig. S2: Strain (MPa) vs Deformation (%) graph for Smooth membrane without been exposed to degradation solution.

Fig. S3: Strain (MPa) vs Deformation (%) graph for Rough membrane without been exposed to degradation solution

Fig. S4: Strain (MPa) vs Deformation (%) graph for Smooth membrane after been exposed to basic degradation solution.

Fig. S5: Strain (MPa) vs Deformation (%) graph for Rough membrane after been exposed to basic degradation solution.

*Fig. S6: Strain (MPa) vs Deformation (%) graph for Smooth membrane after been exposed to acidic degradation solution.*

Fig. S7 Strain (MPa) vs Deformation (%) graph for Rough membrane after been exposed to acidic degradation solution.
